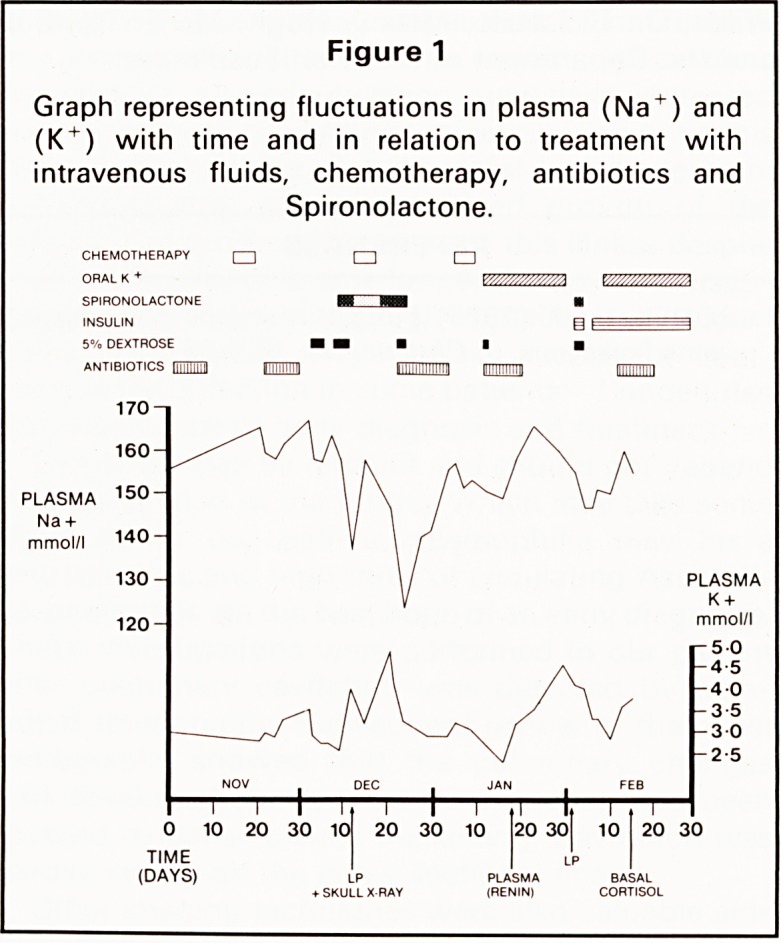# 'Lot's Wife' Syndrome in Acute Myeloid Leukaemia

**Published:** 1984-07

**Authors:** M. M. Barnett

**Affiliations:** Department of Clinical Haematology, Frenchay Hospital, Bristol


### 'Lot's Wife' Syndrome in Acute Myeloid Leukaemia

**M. M. Barnett B.Sc., M.B., B.S.**
Department of Clinical Haematology, Frenchay Hospital, Bristol

### INTRODUCTION

Chronic hypodipsic hypernatraemia in the absence of clinical hypovolaemia, with normal renal function is rare, and has been reported principally in patients with known hypothalamic lesions.[1,2]

Hypernatraemia has been found in acute myeloid leukaemia, but only in association with diabetes insipidus.[3] This appears to be the first reported case of chronic hypodipsic hypernatraemia complicating acute myeloid leukaemia. The title 'Lot's Wife Syndrome' has been coined by the author.[4]

### CASE REPORT

A 49 year old woman presented with lack of energy for 3 months. She had no other symptoms and clinical examination was normal. She weighed 58 kg (height 1 m 60 cm) and her blood pressure was 140/85 mmHg.

A full blood count revealed Hb 11·7 g/dl, WBC 17·5×10⁹/l with 33% blasts in the blood film. A bone marrow trephine confirmed the diagnosis of acute myeloid leukaemia. A random plasma glucose was normal, however, plasma sodium was raised at 154 mmol/l with potassium 3·4 mmol/l, chloride 114 mmol/l, urea 4 mmol/l and creatinine 99 µmol/l.

She had a series of admissions for courses of chemotherapy (daunorubicin, cytosine arabinoside and 6-thioguanine), blood transfusions and treatment of septicaemia associated with neutropaenia (including intravenous cefotaxime and tobramycin). She was otherwise maintained on Augmentin, ketoconazole and allopurinol.

Her plasma electrolytes were persistently deranged (Figure 1). However she had no signs or symptoms of hypernatraemia. She was not thirsty or clinically dehydrated, and had a balanced daily fluid intake and output of 1375–2900 ml, with an estimated dietary intake of sodium 120–150 mmol/day and potassium 65 mmol/day. Her urine sodium output was 80–120 mmol/24 h (random estimations).

Her skull X-ray was normal, as were two lumbar punctures. Morning plasma cortisol of 729 umol/l was suppressed normally by 1 mg Dexamethazone to 256 umol/l.

**Figure 1**

Graph representing fluctuations in plasma ($Na^+$) and ($K^+$) with time and in relation to treatment with intravenous fluids, chemotherapy, antibiotics and Spironolactone.

Plasma renin levels of 25 ng/ml/hr (supine) and 60 ng/ml/hr (erect) suggested secondary hyperaldosteronism, and 75–100 mg spironolactone (an aldosterone antagonist) daily, induced hyponatraemia in 9 days (50 mg was ineffective). However, her blood pressure remained 130/80–90/60 mmHg throughout, with urea mean value 9 mmol/l (range 4·0–16·6) and creatinine mean value 98 µmol/l (range 67–146) and a creatinine clearance of 47 ml/min/m².

Six months after presentation she developed stress hyperglycaemia which responded to insulin therapy, and subsequently resolved as her general condition improved.

Her plasma potassium rose with oral supplements to 3·3–3·6 mmol/l, however her plasma sodium, in the absence of spironolactone, restabilised between 150–159 mmol/l.

Deprivation of water for 13 hr overnight did not make her thirsty, but increased her plasma sodium from 158 to 165 mmol/l with serum osmolality of 357 mOsm/kg and an early morning urine osmolality of 308 mOsm/kg.

### DISCUSSION

Hypodipsic hypernatraemia is uncommon. It has occurred in both neurosurgical and leukaemic patients with diabetes insipidus,[1,2,3] secondary to primary hyperaldosteronism[5] or due to iatrogenic hypertonic saline infusion. In this case, the association with acute myeloid leukaemia suggested hypothalamic infiltration, but no evidence for this was found, nor were there any signs or symptoms of diabetes insipidus. Primary aldosteronism was unlikely, as the patient's blood pressure remained normal or low. At no time was she infused with hypertonic saline.

Hypernatraemia of this type has been ascribed either to partial destruction, or to a resetting, of the hypothalamic osmostat which regulates ADH secretion.

It has been argued[1,2,6] that partial destruction would result in low plasma ADH levels and only a partial response to plasma osmolality, whereas if the osmostat were reset, ADH would be secreted normally, but at a higher threshold plasma osmolality. In this case, plasma ADH levels were not measured, but the fluctuation of plasma sodium concentrations within a limited but elevated range, the response to water deprivation and the matched fluid intake and output, supports the concept of a reset osmostat.

Acknowledgements to Dr. R. D. Eastham for advice and Peter Cox for the illustration.

### REFERENCES

1. DE RUBERTIS, F., MICHELIS, M. F. and DAVIS, B. B. (1974) Essential hypernatraemia. *Arch. Intern. Med.* **134**, 889–895
2. HALTER, J. B., GOLDBERG, A. P., ROBERTSON, G. L. and PORTE, D. Jr. (1977) Selective osmoreceptor dysfunction in the syndrome of hypernatraemia. *J.Endocrinol.Metab.* **44**, 609.
3. NEWCOMER, L. N. (1982) Diabetes insipidus associated with central nervous system leukaemia. *Southern Medical Journal* **75**(9), 1142–1143.
4. HOLY BIBLE. Genesis, Chapter 19, verse 26.
5. GANGULY, A. and ROBERTSON, G. L. (1980). Elevated threshold for vasopressin release in primary aldosteronism. *Clin.Res.* **28**, 330A.
6. ROBERTSON, G. L., AYCINENA P. and ZERBE, R. L. (1982). Neurogenic disorders of osmoregulation— *Am.J.Med.* **72**, 339–353.

## Figures and Tables

**Figure 1 f1:**